# Medium Optimization for Exopolysaccharide Production in Liquid Culture of Endophytic Fungus *Berkleasmium* sp. Dzf12

**DOI:** 10.3390/ijms130911411

**Published:** 2012-09-12

**Authors:** Peiqin Li, Liang Xu, Yan Mou, Tijiang Shan, Ziling Mao, Shiqiong Lu, Youliang Peng, Ligang Zhou

**Affiliations:** 1 Department of Plant Pathology, College of Agronomy and Biotechnology, China Agricultural University, Beijing 100193, China; E-Mails: lipq110@163.com (P.L.); lbaf2006@163.com (L.X.); muyan01987@163.com (Y.M.); shan5400388@163.com (T.S.); maoziling2011@163.com (Z.M.); shiqionglu@126.com (S.L.); pengyl@cau.edu.cn (Y.P.); 2 Department of Forestry Pathology, College of Forestry, Northwest A & F University, Yangling 712100, China

**Keywords:** medium optimization, endophytic fungus *Berkleasmium* sp. Dzf12, exopolysaccharide, fractional factorial design, center composite design, response surface methodology

## Abstract

*Berkleasmium* sp. Dzf12, an endophytic fungus from *Dioscorea zingiberensis*, is a high producer of spirobisnaphthalenes with various bioactivities. The exopolysaccharide (EPS) produced by this fungus also shows excellent antioxidant activity. In this study, the experimental designs based on statistics were employed to evaluate and optimize the medium for EPS production in liquid culture of *Berkleasmium* sp. Dzf12. For increasing EPS yield, the concentrations of glucose, peptone, KH_2_PO_4_, MgSO_4_·7H_2_O and FeSO_4_·7H_2_O in medium were optimized using response surface methodology (RSM). Both the fractional factorial design (FFD) and central composite design (CCD) were applied to optimize the main factors which significantly affected EPS production. The concentrations of glucose, peptone and MgSO_4_·7H_2_O were found to be the main effective factors for EPS production by FFD experimental analysis. Based on the further CCD optimization and RSM analysis, a quadratic polynomial regression equation was derived from the EPS yield and three variables. Statistical analysis showed the polynomial regression model was in good agreement with the experimental results with the determination coefficient (adj-*R*^2^) as 0.9434. By solving the quadratic regression equation, the optimal concentrations of glucose, peptone and MgSO_4_·7H_2_O for EPS production were determined as 63.80, 20.76 and 2.74 g/L, respectively. Under the optimum conditions, the predicted EPS yield reached the maximum (13.22 g/L). Verification experiment confirmed the validity with the actual EPS yield as 13.97 g/L, which was 6.29-fold in comparison with that (2.22 g/L) in the original basal medium. The results provide the support data for EPS production in large scale and also speed up the application of *Berkleasmium* sp. Dzf12.

## 1. Introduction

Recently, extensive attention and interest have been focused on the polysaccharides prepared from fungi for their various biological activities, such as immunomodulating effects of the polysaccharides from *Coriolus versicolor* [1] and *Hericium erinaceus* [2], antioxidant activities of the polysaccharides from *Cordyceps sinensis* [3–5], *Fusarium oxysporum* Dzf17 [6] and *Aspergillus versicolor* [7], antitumor effects of the polysaccharides from *Ganoderma tsugae* [8] and *Pholiota dinghuensis* [9], anti-inflammatory effect of the polysaccharide from *Fomitopsis pinicola* [10], antiherpectin activity of the sulfated polysaccharide from *Agaricus brasiliensis* [11], antiangiogenic activity of the polysaccharide from *Antrodia cinnamomea* [12], anticoagulant properties of the polysaccharides from *Pleurotus sajor*-*caju* [13], and enhancement of diosgenin production in cell suspension culture of *Dioscorea zingiberensis* by the polysaccharides from endophytic fungus *Fusarium oxysporum* Dzf17 [14,15]. Plant endophytic fungi are microorganisms that reside in the internal tissues of living plants without causing any immediate overt negative effects or external symptoms [16]. They have been considered as important and novel potential sources of natural bioactive compounds [17–21]. These bioactive compounds could be classified as alkaloids, terpenoids, steroids, quinones, lignans, phenols, and lactones [22,23]. Most of investigations on fungal polysaccharides mainly focused on higher basidiomycetes mushrooms [24,25]. The polysaccharides from endophytic fungi have been rarely reported except for our previous studies [6,14,15,26].

Endophytic fungus *Berkleasmium* sp. Dzf12 was isolated from the healthy rhizomes of medicinal plant *Dioscorea zingiberensis* [27]. Five spirobisnaphthalenes with antimicrobial activity were isolated from this fungus [28]. It was found that *Berkleasmium* sp. Dzf12 was a high producer of spirobisnaphthalenes [29–32]. Furthermore, three polysaccharides, namely exopolysaccharide (EPS), water-extracted mycelial polysaccharide (WPS) and sodium hydroxide-extracted mycelial polysaccharide (SPS), were prepared from *Berkleasmium* sp. Dzf12, of which EPS showed excellent *in vitro* antioxidant activities by evaluating their DPPH scavenging, reducing Fe^3+^, chelating Fe^2+^ and hydroxyl radical scavenging activities [26]. However, the yield (2.22 g/L) of EPS produced by *Berkleasmium* sp. Dzf12 was low in the original medium [26]. To achieve a high yield of EPS, it is a prerequisite to optimize the medium for EPS production of *Berkleasmium* sp. Dzf12.

Currently, a large number of studies have been reported to optimize the medium for production of desired products in the fermentation process of microorganisms by employing different kinds of statistical experimental design techniques and analytical methods [33–37]. The conventional practice of one-factor-at-a-time method is extremely laborious and time-consuming, and moreover, it does not guarantee the determination of the optimal conditions, and is unable to detect the frequent interactions occurring between two or more factors although they often do occur [38]. The limitations of one-single-factor-experimental optimization process can be eliminated by statistical experimental design combined response surface methodology (RSM), such as factorial design, uniform design, central composite design (CCD) and Box-Behnek design (BBD) [39–42]. In this work, the main effective components in medium for EPS production were firstly determined by a 2^5-1^ fractional factorial design (FFD). And then, CCD experiments and RSM analyses were carried out to optimize the critical factors for realizing the maximization of EPS yield.

## 2. Results and Discussion

### 2.1. FFD Experiments and Statistical Analyses

The fractional factorial design (FFD) enables the identification of the main effect of each variable upon response, which is estimated as the difference between both averages of measurements made at the high and low levels of that factor [36,43]. The impacts of the five factors on EPS production, which were the concentrations (g/L) of glucose, peptone, KH_2_PO_4_, MgSO_4_·7H_2_O and FeSO_4_·7H_2_O, were evaluated by FFD screening experiments. The results of FFD experiments are shown in Table 1, where EPS yield varied markedly from 1.12 to 13.63 g/L. Such a wide variation of EPS yield reflected the potential of parameter optimization to reach higher productivity.

The analysis of variance (ANOVA) of the FFD experiments is summarized in Table 2. By *F*-test analysis of each variable, the concentrations of glucose, peptone and MgSO_4_·7H_2_O were found to have significant effects on EPS production at *p* = 0.01 level, for their low *p*-values (<0.01). While the *p*-values of KH_2_PO_4_ and FeSO_4_·7H_2_O were separately 0.5664 and 0.4641, higher than statistical levels 0.05 or 0.01 [38], which demonstrated the two variables producing not evident effects on EPS production. Hence, the concentrations of glucose, peptone and MgSO_4_·7H_2_O were chosen for further investigation for maximization of EPS production.

### 2.2. Single-Factor Experiments and Analyses

Based on the results and analyses of FFD experiments, the concentrations (g/L) of glucose, peptone and MgSO_4_·7H_2_O in medium were determined as the critical factors on EPS production. Hence, the equally spaced locations of each variable single-factor experiments were carried out to further optimize the three factors, while the concentrations of KH_2_PO_4_ and FeSO_4_·7H_2_O were fixed at 2.0 g/L and 0.05 g/L, respectively.

**Figure 1:**
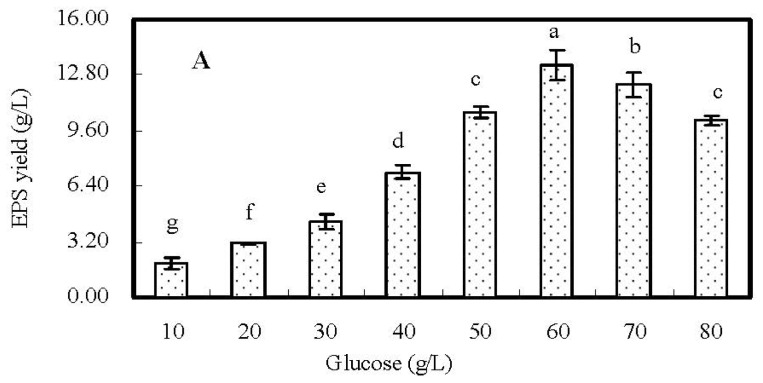

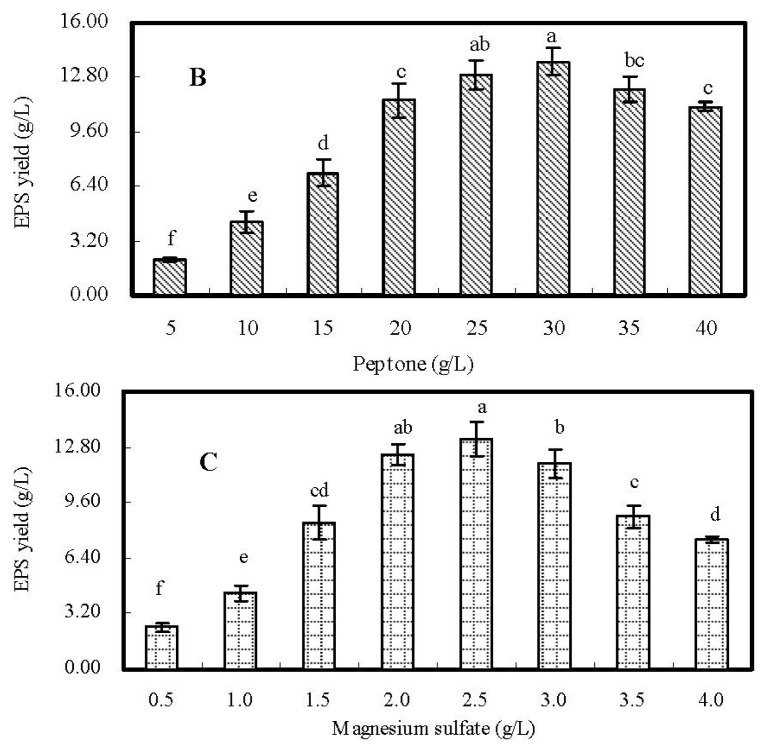
Effects of the concentrations (g/L) of glucose (**A**); peptone (**B**); and MgSO_4_·7H_2_O (**C**) in medium on exopolysaccharide (EPS) production in fermentation culture of *Berkleasmium* sp. Dzf12. The error bars represent standard deviations from three independent samples. Different letters indicate significant differences among the treatments at *p* = 0.05 level.

The effects of the concentration of glucose ranged from 10 to 80 g/L on EPS production are presented in Figure 1A. When the concentration of glucose was increased from 10 to 60 g/L, the EPS yield was obviously increased from 1.98 to 13.37 g/L. However, when the concentration of glucose was higher than 60 g/L, the EPS yield was decreased slightly. It indicated the highest amount of EPS was attained when the concentration of glucose was approximating the neighborhood of 60 g/L. Thus, 60 g/L of glucose was selected as the center point of CCD.

Figure 1B graphs the effects of the concentration of peptone on EPS production in fermentation culture. When the concentration of peptone was increased from 5 to 40 g/L, the EPS yield was significantly. The highest EPS yield (13.69 g/L) was observed when the concentration of peptone was at 30 g/L. Hence, 30 g/L of peptone in medium was chosen as the center point of CCD.

The effects of the concentration of MgSO_4_·7H_2_O on EPS production are shown in Figure 1C. When the concentration of MgSO_4_·7H_2_O varied from 0.5 to 2.5 g/L, the EPS yield was increased from 2.42 to 12.38 g/L. However, when the concentration of MgSO_4_·7H_2_O in fermentation medium was higher than 2.5 g/L, the EPS yield was decreased slightly. It demonstrated the optimal concentration of MgSO_4_·7H_2_O for EPS production was close to 2.5 g/L. Therefore, 2.5 g/L of MgSO_4_·7H_2_O was selected as the center point of CCD.

### 2.3. CCD Experiments, Model Building and Statistical Analysis

According to the results of FFD and single-factor experiments, the suitable concentrations of glucose, peptone and MgSO_4_·7H_2_O in medium for EPS production were determined for further CCD experiments. Five levels of each variable were set by software of Design Expert, which are presented in Table 3. And then 20 trials of CCD were carried out to optimize the production of EPS. The results of CCD experiments were summarized in Table 4. The EPS yield displayed a considerable variation from 2.71 to 13.43 g/L depending upon the changes of variables. Based on the results of CCD experiments, a second-order polynomial regression model between EPS yield and the tested independent variables was derived by software of Design Expert as follows (Equation 1):

(1) $$Y=12.52+1.30x_{1}+1.12x_{2}+1.51x_{3}-1.06x_{1}x_{2}+1.15\;x_{1}x_{3}-0.50x_{2}x_{3}-2.24{x_{1}}^{2}-1.56{x_{2}}^{2}-1.93{x_{3}}^{2}$$

in the Equation, *Y* represented the EPS yield (g/L), and *x*_1_, *x*_2_ and *x*_3_ were the coded values of the test variables, the concentrations (g/L) of glucose, peptone and MgSO_4_·7H_2_O.

In order to determine whether the quadratic regression model was significant or not, the ANOVA analysis was conducted, which is summarized in Table 5. The ANOVA of the quadratic regression model demonstrated that the model was highly significant, evident from the Fisher’s *F*-test with a very high model *F*-value (78.46) but a very low *p*-value (*p* < 0.0001). The goodness of the model was examined by the determination coefficients (*R*^2^) and the multiple correlation coefficients (*R*). The value of the determination coefficient adj-*R*^2^ (0.9434) demonstrated that the total variation of 94.34% for EPS yield was attributed to the tested independent variables and only about 5.66% of the total variation could not be explained by the model. The value of *R* was closer to 1, the fitness of the model was better [44]. In this research, the multiple correlation coefficients adj-*R* of the model was 0.9712, indicating a good agreement between the experimental and predicted values. As presented in Table 4, the differences between the experimental and predicted EPS yields for the 20 trials of CCD were dramatically small, nearly close to zero. The lack-of-fit measured the failure of the model to represent the data in the experimental domain at points which were not included in the regression [45]. The *F*-value for lack-of-fit was 0.59 and the corresponding *p*-value was 0.71 (>0.05), which implied the lack-of-fit was not significant relative to the pure error due to noise. Insignificant lack-of-fit confirmed the validity of the model.

The coefficients of the quadratic polynomial model, along with their corresponding *p*-values, are calculated and presented in Table 6. The *p*-value was used as a tool to check the significance of each coefficient, which also indicated the interaction strength between each independent parameter [46]. The smaller the *p*-value was, the bigger the significance of the corresponding coefficient should be [47]. It can be seen from Table 6 that all regression coefficients of the quadratic polynomial model were highly significant with low *p*-values.

### 2.4. Response Surface and Contour Plots Analyses

**Figure 2:**
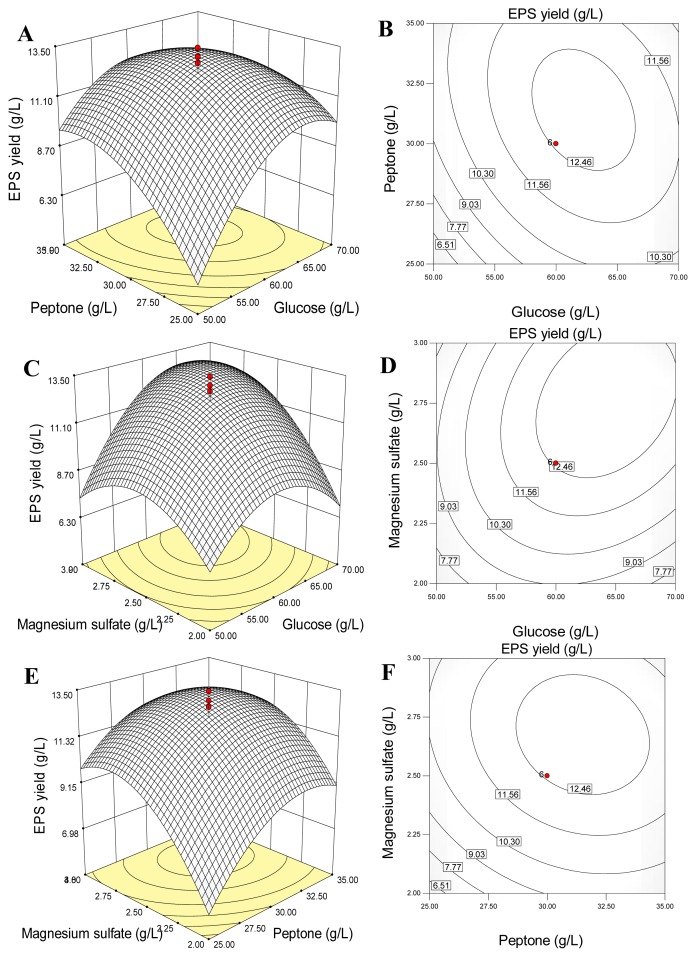
The 3D-response surface and 2D-contour plots of EPS yield (g/L) *versus* the tested variables (g/L): glucose and peptone (**A**,**B**); glucose and MgSO_4_·7H_2_O (**C**,**D**); peptone and MgSO_4_·7H_2_O (**E**,**F**).

The three-dimensional (3D) response surface and two-dimensional (2D) contour plots are the graphical representations of the quadratic polynomial regression equation [48]. They provide a method to visualize the relationship between the responses and the experimental levels of each variable, and the interactions between any two tested variables from the circular or elliptical nature of contour [49]. A circular contour plot indicates that the interactions between the corresponding variables are negligible. An elliptical nature of the contour plots indicates that the interactions between the corresponding variables are significant [50]. In the present study, the 3D response surfaces and 2D contour plots are presented in Figure 2, which were generated by employing the software of Design-Expert. Analyses of the 3D response surfaces and their corresponding 2D contour plots allowed us to conveniently investigate the interactions between any two variables, and locate the optimum ranges of the variables efficiently such that the response was maximized. The maximum predicted response was indicated by the surface confined in the smallest ellipse in the contour diagram.

The response surface plot in Figure 2A and contour plot in Figure 2B show the effects of glucose and peptone on EPS yield and their interactions when MgSO_4_·7H_2_O was fixed at zero level. EPS yield showed an increasing tendency with the increasing of the concentrations of glucose and peptone, and then decreased slightly. A full elliptic contour in Figure 2B was observed, indicating a significant interaction between glucose and peptone for EPS production. It was consistent with the analyses of coefficients of the regression equation (Table 6). Figure 2C,D graphed the effects of glucose and MgSO_4_·7H_2_O on EPS yield and their interaction when peptone was fixed at zero level. When the concentrations of glucose and MgSO_4_·7H_2_O in medium were increased from the lowest levels to the highest levels, EPS yield was increased initially and then decreased. The elliptic contour in Figure 2D indicated the significant interaction between glucose and MgSO_4_·7H_2_O for EPS production. The effects of peptone and MgSO_4_·7H_2_O, and their interactions, on EPS yields are shown in Figure 2E,F. EPS yield was firstly augmented and then decreased when the concentrations of peptone and MgSO_4_·7H_2_O varied from the lowest levels to the highest levels.

By analyzing the 3D response surface and 2D contour plots, the corresponding point to the maximum of EPS yield should locate on the peak of the response surface, which projected in the smallest ellipse in the contour diagram [51]. Hence, the optimal ranges of the concentrations of glucose, peptone and MgSO_4_·7H_2_O in medium for realizing the maximization of EPS yield were calculated by the software Design Expert as follows: 58.30 to 66.48 g/L for glucose, 28.89 to 33.93 g/L for peptone, 2.44 to 2.93 g/L for MgSO_4_·7H_2_O.

### 2.5. Optimization of the Variables and Verification of the Model

By solving the inverse matrix of the regression polynomial equation (Equation 1) employing the software of Design-Expert, the optimum values of the tested parameters in uncoded units were obtained as follows: glucose as 63.80 g/L, peptone as 20.76 g/L, and MgSO_4_·7H_2_O as 2.74 g/L. Under the optimum conditions, the predicted EPS yield reached to the maximum (13.22 g/L). To validate the suitability of the model equation for predicting the optimum response value, experimental rechecking was performed using the deduced optimal conditions. Under the determined conditions, a mean value of EPS yield of 13.97 g/L (*n* = 5) was obtained from the actual experiments, slightly higher than the predicted maximum value (13.22 g/L). However, no significant difference was observed between the predicted yield and experimental one when the Student *t*-test was conducted, indicating that the model was satisfactory and adequate for reflecting the expected optimization.

## 3. Experimental Section

### 3.1. Cultivation of the Endophytic Fungus *Berkleasmium* sp. Dzf12

The endophytic fungus *Berkleasmium* sp. Dzf12 (GenBank accession number EU543255) was isolated from the healthy rhizomes of *D. zingiberensis* in our previous study [27,28]. It was preserved on potato dextrose agar (PDA) slants at 4 °C and subcultured every six months.

*Berkleasmium* sp. Dzf12 was firstly cultivated in a 150-mL flask containing 30 mL modified Sabouraud broth medium consisting of glucose (40 g/L), peptone (10 g/L), KH_2_PO_4_ (1.0 g/L), MgSO_4_·7H_2_O (0.5 g/L), FeSO_4_·7H_2_O (0.05 g/L), which was incubated at 25 °C on a rotary shaker at 150 rpm for 4 days as the inoculated seed culture [29]. The initial pH of culture medium was adjusted to 6.5. The culture medium producing EPS was confected according to the experimental design based on the basic medium composed of glucose, peptone, KH_2_PO_4_, MgSO_4_·7H_2_O and FeSO_4_·7H_2_O. The medium composition (g/L) was set according to the experimental design. Each 250-mL Erlenmeyer flask containing 100 mL of fermentation medium was inoculated with 2.5% (*v*/*v*) seed culture broth, then cultivated in a rotary shaker incubator at 25 °C, 150 rpm for 12 days [29].

### 3.2. Preparation of the Exopolysaccharide

Exopolysaccharide (EPS) was prepared from fermentation broth of *Berkleasmium* sp. Dzf12 according to our previous reports [14,26]. The 12-day-old *Berkleasmium* sp. Dzf12 fermentation broth was harvested and centrifugated. The supernatant without mycelia was collected and concentrated to a proper volume (about 10% of the original) under vacuum at 60 °C by a rotary evaporator and mixed with three volumes of 95% ethanol. The mixture was stirred vigorously and then maintained at 4 °C for 48 h. The precipitate was collected by centrifugation at 17,418*g* for 15 min from the ethanol dispersion and then washed twice with absolute ethanol and acetone respectively. The final precipitate was then subjected to successive deproteination with Sevag reagent (chloroform-*n*-butanol at 4:1, *v*/*v*), decolorization with H_2_O_2_, and removal of small molecular impurities by dialysis. Polysaccharide mixture with molecular weight greater than 8000–14,000 Da was kept in the dialysis tube. The retentate was concentrated to a certain volume and then mixed with three volumes of 95% ethanol. The precipitate thus obtained was lyophilized and weighed, which was designated as EPS.

The carbohydrate content of EPS was measured spectrophotometrically by the method of anthrone-sulfuric acid [15,52], which involved sulfuric acid hydrolysis of the sample in the presence of anthrone agent at 100 °C. The absorbance at 620 nm was measured and calibrated to carbohydrate content using glucose as a reference. The EPS yield was calculated by the amount (g) of carbohydrate content of EPS per liter (L) culture medium.

### 3.3. Procedure Optimization and Experimental Design

Fractional factorial design (FFD) was initially employed to identify the major components of medium affecting the producing of EPS, which was very practical, especially when the investigator is faced with a large number of factors and is unsure which settings are likely to be close to optimum responses [33,53]. Five components of the fermentation medium (glucose, peptone, KH_2_PO_4_, MgSO_4_·7H_2_O and FeSO_4_·7H_2_O) were selected as factors to conduct the 2^5-1^ FFD experiments using the software of Design Expert (Version 7.1; Stat-Ease, Inc.: Minneapolis, MN, USA). Each factor was set at a high level (coded + 1) and a low level (coded − 1), which are listed in Table 7. The FFD matrix is shown in Table 1 including 16 runs. All experiments were conducted in triplicate and the averages of the results were taken as response values.

By analyzing the results of factorial design experiments, three main components having significant effects on EPS production were determined as glucose, peptone and MgSO_4_·7H_2_O. Single-factor experiments of the three major factors were carried out to determine their optimal ranges for EPS production, when the concentrations of KH_2_PO_4_ and FeSO_4_·7H_2_O were fixed at 2.0 g/L and 0.05 g/L, respectively.

Based on the results of fractional factorial and single-factor experiments, central composite design (CCD) experiments and response surface methodology (RSM) were employed to optimize the concentrations of glucose, peptone and MgSO_4_·7H_2_O in the fermentation medium for realizing the maximization of EPS yield by the software of Design-Expert. In recent years, both CCD and RSM technologies have been widely applied to optimize the medium composition for production of different metabolites from fungi, which have also been proved to be efficient, practical and precise [54,55]. Each independent variable in the CCD experiments was studied at five levels (−1.682, −1, 0, +1, +1.682), which is represented in Table 3. The independent variable was expressed as *X*_i_, which was coded as *x_i_* according to the following equation (Equation 2):

(2) $$x_{i}=(X_{i}-X_{0})/\Delta X,\;i=1,2,3$$

where *x_i_* is the coded value of the variable *X_i_*, while *X*_0_ is the value of *X_i_* at the center point, and Δ*X* is the step change of an independent variable.

CCD in this experimental design consisted of 20 trials which were carried out in a random order in triplicate that was necessary to estimate the variability of measurements, which are presented in Table 4. Five replicates at the center point of the design were carried out to allow for estimation of a pure error sum of squares. The EPS yield was recorded as the mean of triplicates, which was taken as the response value.

Based on the CCD experimental data, a second-order polynomial model was established, which correlated the relationship between EPS yield and the independent variables. The relationship could be expressed by the following equation (Equation 3):

(3) $$Y={\text{a}}_{0}+{\text{a}}_{1}x_{1}+{\text{a}}_{2}x_{2}+{\text{a}}_{3}x_{3}+{\text{a}}_{12}x_{1}x_{2}+{\text{a}}_{13}x_{1}x_{3}+{\text{a}}_{23}x_{2}x_{3}+{\text{a}}_{11}{x_{1}}^{2}+{\text{a}}_{22}{x_{2}}^{2}+{\text{a}}_{33}{x_{3}}^{2}$$

where *Y* is the predicted response value; a_0_ is the intercept term; *x*_1_, *x*_2_ and *x*_3_ are independent variables; a_1_, a_2_ and a_3_ are linear coefficients; a_12_, a_13_ and a_23_ are cross product coefficients; and a_11_, a_22_ and a_33_ are the quadratic term coefficients. All of the coefficients of the second polynomial model and the responses obtained from the experimental design were subjected to multiple nonlinear regression analyses.

The fitness of the second-order polynomial model equation was evaluated by the coefficient (*R*^2^) of determination. The analysis of variance (ANOVA) and test of significance for regression coefficients were conducted by *F*-test. In order to visualize the relationship between the response values and independent variables, the fitted polynomial equation was separately expressed as 3D response surfaces and 2D contour plots by the software of Design Expert [56,57].

## 4. Conclusions

The medium composition (*i.e.*, the concentrations of glucose, peptone, KH_2_PO_4_, MgSO_4_·7H_2_O and FeSO_4_·7H_2_O) for EPS production of *Berkleasmium* sp. Dzf12 was optimized in this study by employing statistical method based on the response surface methodology (RSM). The FFD experiments were initially carried out to screen the main effective factors, and the three variables (*i.e*., the concentrations of glucose, peptone and MgSO_4_·7H_2_O) were found to have significant impacts on EPS production. Single-factor experiments were further conducted to define the optimal ranges of the three main variables and to provide basis for the later CCD experiments. Both CCD experiments and RSM technology were applied to optimize the concentrations of glucose, peptone and MgSO_4_·7H_2_O in medium to realize the maximization of EPS yield. By solving the quadratic regression equation between EPS yield and the three variables, the optimal concentrations of glucose, peptone and MgSO_4_·7H_2_O in medium were determined as 63.80, 20.76 and 2.74 g/L, respectively. Under the optimum conditions, the predicted EPS yield reached the maximum (13.22 g/L). The predicted EPS yield showed no significant difference from the experimental value. By optimizing the medium of *Berkleasmium* sp. Dzf12 for EPS production, the EPS yield was increased to 13.97 g/L, which was 6.29-fold in comparison with that (2.22 g/L) in the original basal medium. The results should be beneficial for future EPS production in fermentation culture of *Berkleasmium* sp. Dzf12 as well as for speeding up the EPS investigation and application. Medium optimization for spirobisnaphthalenes (*i.e.*, palmarumycins C_12_ and C_13_) production in mycelial liquid culture of *Berkleasmium* sp. Dzf12 has been studied in our previous studies [29–32]. As there were only five components in the medium used in this study, more components in the medium as well as other parameters like pH, temperature, oxygen supply, and ionic strength should be considered in future work. Optimization of the medium to produce both spirobisnaphthalenes and EPS with their high yields, as well as consideration of the medium viscosity and foam formation in a bioreactor are other aspects worthy of further study.

## Figures and Tables

**Table 1:** The matrix of fractional factorial design (FFD) and the experimental results.

Run	Glucose (g/L)	Peptone (g/L)	KH_2_PO_4_ (g/L)	MgSO_4_·7H_2_O (g/L)	FeSO_4_·7H_2_O (g/L)	EPS Yield (g/L)
1	30	10	0.5	0.5	0.05	1.20
2	60	10	0.5	0.5	0.01	1.42
3	30	20	0.5	0.5	0.01	2.80
4	60	20	0.5	0.5	0.05	3.67
5	30	10	2.0	0.5	0.01	1.12
6	60	10	2.0	0.5	0.05	2.36
7	30	20	2.0	0.5	0.05	3.85
8	60	20	2.0	0.5	0.01	6.62
9	30	10	0.5	2.0	0.05	2.82
10	60	10	0.5	2.0	0.01	5.37
11	30	20	0.5	2.0	0.01	5.08
12	60	20	0.5	2.0	0.05	13.63
13	30	10	2.0	2.0	0.01	2.97
14	60	10	2.0	2.0	0.05	6.84
15	30	20	2.0	2.0	0.05	5.57
16	60	20	2.0	2.0	0.01	10.89

**Table 2:** Analysis of variance (ANOVA) of the fractional factorial design (FFD) experiments.

Source	Sum of squares	d.f.	*F* Value	*p-*Value *p* > *F*	Significance
Glucose	40.29	1	12.67	0.0052	**
Peptone	49.04	1	15.42	0.0028	**
KH_2_PO_4_	1.12	1	0.35	0.5664	
MgSO_4_·7H_2_O	56.74	1	17.84	0.0018	**
FeSO_4_·7H_2_O	1.84	1	0.58	0.4641	

** significance of the variable: *p* = 0.01.

**Table 3:** Coded values (*x*) and uncoded values (*X*) of variables in the central composite design (CCD) experiments.

Variable (g/L)	Symbol		Coded level				
	
	Uncoded	Coded	−1.682	−1	0	1	+1.682
Glucose	*X*_1_	*x*_1_	43.18	50	60	70	76.82
Peptone	*X*_2_	*x*_2_	21.59	25	30	35	38.41
MgSO_4_·7H_2_O	*X*_3_	*x*_3_	1.66	2	2.5	3	3.34

**Table 4:** CCD experimental matrix and the results.

				EPS Yield (g/L)		
				
Run	*x*_1_	*x*_2_	*x*_3_	Experimental *Y*_e_	Predicted *Y*_p_	*Y*_e_ − *Y*_p_
1	0	−1.682	0	6.07	6.23	−0.17
2	0	0	1.682	9.30	9.59	−0.29
3	0	1.682	0	10.22	9.99	0.23
4	−1	−1	−1	2.71	2.45	0.26
5	−1	1	1	7.89	7.52	0.36
6	−1	1	−1	7.32	7.81	−0.49
7	1	1	1	10.01	10.31	−0.30
8	0	0	0	13.43	12.52	0.91
9	0	0	0	11.95	12.52	−0.57
10	1	−1	−1	4.47	4.88	−0.41
11	1	1	−1	6.01	6.00	0.01
12	0	0	−1	4.87	4.52	0.35
13	1.682	0	0	8.51	8.39	0.13
14	−1	−1	1	4.12	4.18	−0.06
15	0	0	0	12.09	12.52	−0.43
16	0	0	0	12.73	12.52	0.21
17	−1.682	0	0	3.94	4.00	−0.06
18	1	−1	1	11.64	11.20	0.44
19	0	0	0	13.02	12.52	0.50
20	0	0	0	11.89	12.52	−0.43

**Table 5:** Analysis of variance (ANOVA) for the fitted quadratic polynomial model.

Source	Sum of squares	d.f.	Mean square	*F* Value	Probability *p* > *F*
Model	228.02	9	25.34	78.46	<0.0001
Lack of fit	1.20	5	0.24	0.59	0.71
Pure error	2.03	5	0.41		
Corrected total	231.25	19			

*R*^2^ = 0.9735; adj-*R*^2^ = 0.9434; *R* = 0.9867; adj-*R* = 0.9712; CV (%) = 6.60.

**Table 6:** Regression coefficient and their significance test of the quadratic polynomial model.

Model term	Coefficient estimate	Standard error	Sum of squares	d.f.	Mean square	*F* Value	Probability *p* > *F*
Intercept	12.52	0.23					
*x*_1_	1.30	0.15	23.18	1	23.18	71.79	<0.0001
*x*_2_	1.12	0.15	17.06	1	17.06	52.83	<0.0001
*x*_3_	1.51	0.15	31.12	1	31.12	96.36	<0.0001
*x*_1_*x*_2_	−1.06	0.20	8.96	1	8.96	27.75	<0.0001
*x*_1_*x*_3_	1.15	0.20	10.58	1	10.58	32.76	0.0004
*x*_2_*x*_3_	−0.50	0.20	2.01	1	2.01	6.24	0.0002
*x*_1_^2^	−2.24	0.15	72.04	1	72.04	223.09	0.0316
*x*_2_^2^	−1.56	0.15	34.99	1	34.99	108.36	<0.0001
*x*_3_^2^	−1.93	0.15	53.80	1	53.80	166.61	<0.0001

**Table 7:** The coded and actual values in the 2^5-1^ FFD experiments.

Variable (g/L)	Level	

	−1	+1
Glucose	30	60
Peptone	10	20
KH_2_PO_4_	0.5	2.0
MgSO_4_·7H_2_O	0.5	2.0
FeSO_4_·7H_2_O	0.01	0.05
